# Risk Assessment and Meningococcal A Conjugate Vaccine Introduction in Africa: The District Prioritization Tool

**DOI:** 10.1093/cid/civ671

**Published:** 2015-11-09

**Authors:** Laurence Cibrelus, Clément Lingani, Katya Fernandez, Mamoudou H. Djingarey, William A. Perea, Stéphane Hugonnet

**Affiliations:** 1 Department of Pandemic and Epidemic Diseases, World Health Organization, Geneva, Switzerland; 2 Inter-country Support Team for West Africa, World Health Organization, Ouagadougou, Burkina Faso

**Keywords:** meningococcal meningitis, PsA-TT, sub-Saharan Africa, risk assessment, vaccine introduction

## Abstract

***Background.*** A group A meningococcal (MenA) conjugate vaccine has progressively been introduced in the African meningitis belt since 2010. A country-wide risk assessment tool, the District Prioritization Tool (DPT), was developed to help national stakeholders combine existing data and local expertise to define priority geographical areas where mass vaccination campaigns should be conducted.

***Methods.*** DPT uses an Excel-supported offline tool that was made available to the countries proposed for immunization campaigns. It used quantitative–qualitative methods, relying predominantly on evidence-based risk scores complemented by expert opinion.

***Results.*** DPT was used by most of the countries that introduced the group A conjugate vaccine. Surveillance data enabled the computation of severity scores for meningitis at the district level (magnitude, intensity, and frequency). District data were scaled regionally to facilitate phasing decisions. DPT also assessed the country's potential to conduct efficient preventive immunization campaigns while paying close attention to the scope of the geographic extension of the campaigns. The tool generated meningitis district profiles that estimated the number of vaccine doses needed. In each assessment, local meningitis experts contributed their knowledge of local risk factors for meningitis epidemics to refine the final prioritization decisions.

***Conclusions.*** DPT proved to be a useful and flexible tool that codified information and streamlined discussion among stakeholders while facilitating vaccine distribution decisions after 2011. DPT methodology may be tailored to prioritize vaccine interventions for other diseases.

Predominantly caused by *Neisseria meningitidis* group A (NmA), recurrent epidemics of meningococcal meningitis have placed a heavy toll on countries of the African “meningitis belt” [1–7]. Until recently, the combination of early detection, case management, and reactive immunization using polysaccharide vaccines were the sole approaches available to control these epidemics. Logistically intensive, this strategy did not alleviate the burden of meningitis in the long term [8–11]. A group A meningococcal polysaccharide–tetanus toxoid conjugate vaccine (PsA–TT) was developed to eliminate NmA epidemics in the most affected countries. PsA-TT is safe and induces a strong and persistent immunity against NmA, preventing carriage and inducing herd protection [12].

PsA-TT has been progressively introduced in the meningitis belt since 2010 through mass immunization campaigns, following the approval of a World Health Organization (WHO)/United Nations Children's Fund (UNICEF) investment case to the Gavi Alliance [10, 13]. By 2016, it is expected that >450 million people in 26 countries at risk for meningitis epidemics in sub-Saharan Africa will be preventively protected against NmA. Countries were grouped according to meningitis risk across the belt, the global vaccine supply (50–70 million doses), and the funds available to support mass immunization campaigns [10, 13]. Burkina Faso, Mali, and Niger were identified as hyperendemic countries and country-wide campaigns in these 3 countries were completed by the end of 2011 [13, 14]. For the other meningitis belt countries, inter- and intracountry risk assessments were required to identify areas of high priority for vaccination.

The District Prioritization Tool (DPT) for NmA immunization was developed to standardize meningitis risk assessment, to define vaccine demand forecasts from 2011 onward, to reinforce in-country advocacy for preventive campaigns, and to support country planning and funding applications. DPT was a decision-making tool that optimized the use of the available country data while ensuring that expert opinion and local knowledge were included into the final decision-making process. Special efforts were made to integrate countries’ ability to mount immunization campaigns [15, 16]. This article describes the tool and presents analytic examples in diverse settings.

## METHODS

### Principles

**Figure 1.**
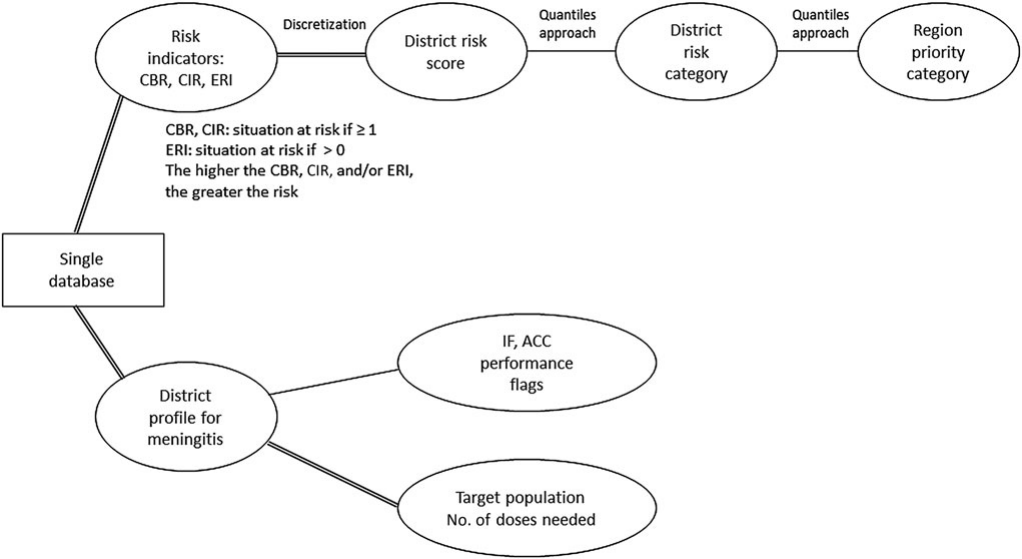
Summary of quantitative phase of District Prioritization Tool evaluation. Abbreviations: ACC, ability to conduct the campaigns; CBR, case burden ratio; CIR, cumulative incidence ratio; ERI, epidemic risk index; IF, immunity front; 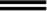, automatic computation; 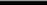, semiautomatic computation (interpretation required).

DPT is an Excel-supported offline tool available in English and French (CD-ROM, also accessible at the WHO website [17]). It assesses the risk of NmA meningitis at the district level—the geographical unit used for meningitis standardized surveillance and control throughout the meningitis belt—in up to 850 districts in any given country [3, 11]. Figure 1 provides an overview of the tool, which is made of 7 connected worksheets, each addressing an aspect of the evaluation: information; data entry; risk indicators (RIs); risk scores (RSs), ranks, and categories; regional scale-up; performance flags; and district profiles. To support planning for vaccine introduction and applications for funding, the Ministry of Health (MOH) in each country used the tool to conduct a national meningitis risk assessment, which included input from national experts in surveillance and immunization and a team of WHO experts.

### Input Variables and Data Collection

Meningitis risk factors were identified by reviewing available data and canvassing expert knowledge [2, 18–22]. Selected variables included the NmA epidemic risk, case burden, and an estimate of immunity following immunization (assuming a 3-year protection by polysaccharide vaccines) or previous epidemics. Additional variables included an assessment of the country's ability to conduct preventive immunization campaigns. In addition, consideration was taken to maintain a geographic continuity of the mass campaigns as they extended out from Burkina Faso, Mali, and Niger (Table 1). Existing standards for meningitis belt surveillance were used [3, 11]. For each risk assessment, aggregated data were collected from official MOH records, from WHO country offices, and from other institutional partners. Data from published and unpublished literature were also collected when available. Data quality was controlled by comparing data from different sources, with reliability judged by local experts and priority given to official records. This information was entered into the corresponding DPT worksheets.

**Table 1.** Variables Used for Quantitative Risk Analysis and Attribution of Performance Flags With the District Prioritization Tool for *Neisseria meningitidis* Serogroup A Vaccination

Denominators	Number of districts in the country Number of regions Number of years of standardized surveillance (ie, study period) Population
Epidemiological risk	Number of meningitis suspected cases^a,b^ Yearly incidence^b^ Identification of NmA^a^ Number of outbreaks due to NmA since standardized surveillance^a,b^ Number of years since last NmA outbreak^b^ Number of reactive immunization campaigns against NmA over the previous 3 years (using polysaccharide vaccine)^b^ Average vaccine coverage reached after reactive immunization against NmA over the previous 3 years^b^
Case burden	Total number of NmA cases prior to and after standardized surveillance^a,b^ Proportion of total number of cases in the country^a,b^
Performance flags	Immunity front: existence of a border with a district already immunized with PsA-TT^b^ Ability to conduct the campaign: Number of preventive mass immunization campaigns for other diseases than meningitis conducted in each district over the 3 previous years by the Ministry of Health and partners (eg, yellow fever, measles)^b^ and average vaccine coverage reached per district after these campaigns^b^
Population information	Total population Target population (70% of total population) Number of doses based on wastage rate Population density^b^

Abbreviations: NmA, *Neisseria meningitidis* group A; PsA-TT, group A meningococcal polysaccharide–tetanus toxoid conjugate vaccine.

^a^ Per district per year over study period.

^b^ Also presented in the district profiles for meningitis.

### Risk Indicators

Three RIs were created to gauge the district meningitis severity level: (1) the case burden ratio was assessed by the proportion of cases in the district weighted by the total number of districts (CBR = district cumulative number of cases divided by country total number of the cases, multiplied by total number of districts in the country); (2) intensity was assessed by the cumulated incidence ratio (CIR = cumulative attack rate in the district divided by country attack rate); and (3) epidemic frequency was described using the epidemic risk index (ERI = annual average number of NmA epidemics over the period of interest), where an epidemic was defined as a yearly district attack rate >100 cases per 100 000 population with identification of NmA at the district level at least once during a given year [8].

### Outputs: Risk Scores and Categories, Risk Mapping, Performance Flags, and District Profiles

Three RSs are automatically generated by the DPT, based on RI values obtained for each district. These scores corresponding to RI values were defined when setting up the tool, through an exploratory analysis of 12-year national datasets from 2 meningitis belt countries, Niger and Burkina Faso. In this analysis, RI values were discretized based on their plotted distribution for all districts in the 2 countries: 5 and 4 categories were created for CBR/CIR and for ERI, respectively, including a “zero risk” category for each RI (<1 for CBR/CIR and 0 for ERI). Quartiles (CBR/CIR) and terciles (ERI) of nonzero values were calculated and attributed a score equal to the quarter (CBR/CIR) or the third (ERI) of a 0–100 scale (ie, with score increments of 25 [CBR/CIR] or 33 [ERI] per category) (Table 2) [23, 24].

**Table 2.** Values of District Prioritization Tool Risk Indicators and Corresponding Risk Scores

Risk Indicator		Scale				
CBR (case burden ratio)	Value	<1	1–2.9	3–4.9	5–9.9	≥10
Score		0	25	50	75	100
CIR (cumulated incidence ratio)	Value	<1	1–1.9	2–5.9	6–19.9	≥20
Score		0	25	50	75	100
ERI (epidemic risk index)	Value	0	0.1–0.2	0.3–0.4	≥0.5	
Score		0	33	66	100	

Abbreviations: CBR, case burden ratio; CIR, cumulative incidence ratio; ERI, epidemic risk index.

The tool also calculates a total RS per district (sum of CBR, CIR, and ERI scores) to rank districts by risk level and define “very high,” “high,” and “moderate” risks using quantiles principles applied to nonzero total RSs. When the total RS is null, the risk is “low” (Figure 1).

Whenever a country needs to conduct the immunization campaigns in phases, district-level outputs are scaled up to the regional level. The proportions of districts of “very high” and “high” risks and the average district RS are computed for each region to rank them. Regions with an average RS equal to zero are given a “low” priority and regions with nonzero average RSs are categorized as “very high,” “high,” and “moderate” priority using quantile principles as well (Figure 1). Districts' risk level and regions' category of priority are mapped using geographic information system software.

In addition to the risk evaluation parameters, the opportunity to expand the geographic area of NmA immunity (called the immunity front) in the population and the ability to implement a large vaccination campaign were also assessed by adding so-called ”performance flags.” The immunity front (IF) performance flag was given to districts bordering an area that had used PsA-TT, while the campaign (ACC) performance flag was given to districts that would not require additional logistical support and planning to implement the immunization campaigns and had successfully mounted preventive mass immunization campaigns conducted over the 3 previous years. Districts reporting coverage rates >80% for poliomyelitis or 90% for measles and/or yellow fever were given an ACC flag [25–27]. DPT summarized the information necessary to attribute the performance flags (Table 1). Districts given an IF and/or ACC flags are listed on the corresponding worksheet of the tool, and the flags (IF or ACC) were added to the risk maps.

DPT also generated “district profiles for meningitis”—that is, a set of figures and histograms that summarize meningitis incidence since the implementation of standardized surveillance, case burden, and history of NmA outbreaks. Using estimated target population, the corresponding number of doses for various wastage rates [10, 13, 14] (Table 1) was computed.

### Expert Opinion

During conferences, all findings were submitted to MOH, national meningitis experts, and decision makers in each country. This step ensured the ownership of the stakeholders in the assessment and priority phasing while taking advantage of the special knowledge of local experts to assure that a tailored introduction strategy for PsA-TT was the final result.

## RESULTS

### Input Variables and Data Collection

**Figure 2.**
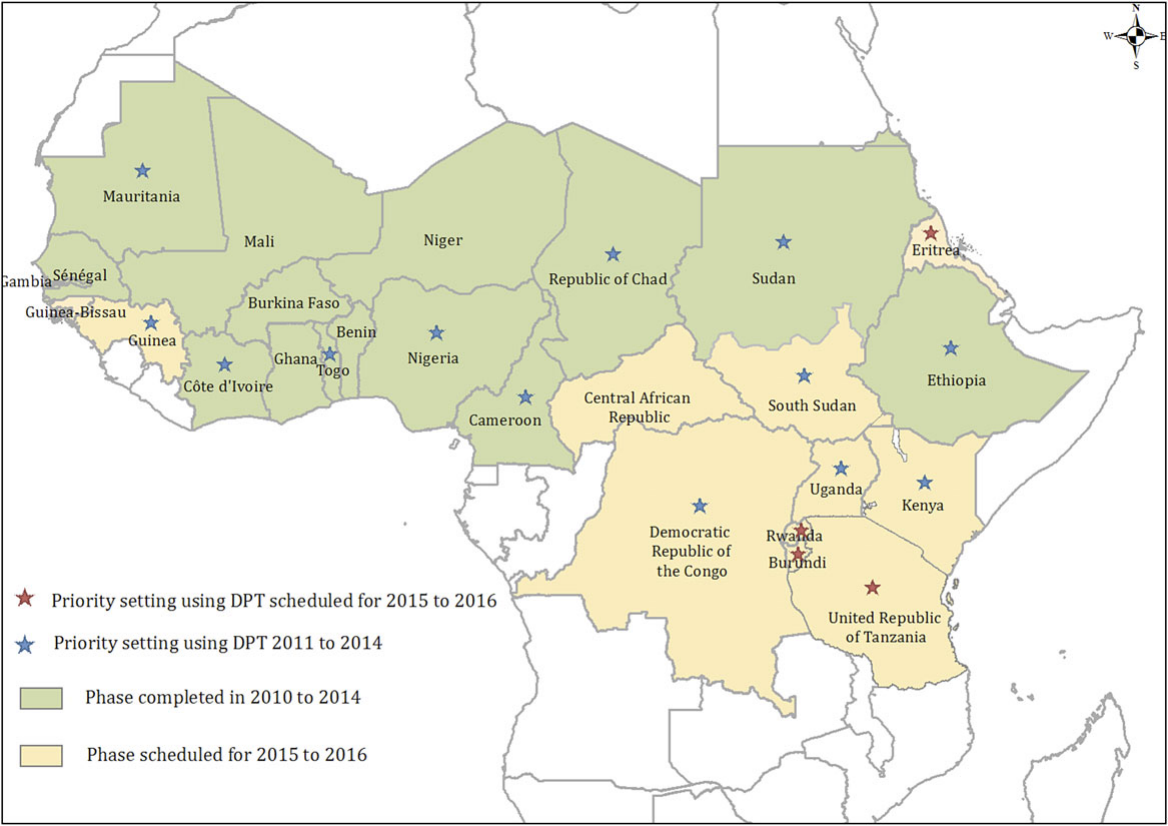
Progress of group A meningococcal polysaccharide–tetanus toxoid conjugate vaccine (PsA-TT) conjugate vaccine introduction and District Prioritization Tool (DPT) evaluations in Africa, 2010–2016.

As of December 2014, DPT evaluations were conducted in 13 of the 17 countries (76%) proposed for PsA-TT mass campaigns. Evaluations are being planned in the remaining countries at risk for meningitis epidemics (Figure 2). DPT was piloted and refined in July and August 2011 in Nigeria, Chad, and Cameroon. Key aspects of the 13 evaluations performed in 2011–2014 are summarized in Table 3. Aggregated data were obtained from the national surveillance systems known as “meningitis epidemic enhanced surveillance” or “integrated disease surveillance and response” [28].

**Table 3.** Metadata and Outcome of District Prioritization Tool Evaluations by Chronological Order, Africa, July 2011–December 2014

Country	Year of DPT Evaluation	Scale of Primary Evaluation	Scale of Campaign Implementation	Surveillance Years Included	Estimated Target Population at the Time of DPT Evaluation	Proposed No. of Phases for PsA-TT Introduction
Nigeria^a^	2011	LGA (353)	State (26)	2007–2011	50 547 012	3
Chad^a^	2011	District (61)	Delegation (20)	2006–2011	5 113 785	2
Cameroon^a^	2011	District (179)	Region (10)	2006–2011	6 012 450	2
Sudan	2011	Locality (156)	State (15)	2005–2011	24 787 935	2
Ethiopia	2012	Zone (97)	Region (11)	2004–2011	58 427 373	3
DRC^b^	2012	Zone (515)	District (42) or province (11)	2005–2012	NA	NA
South Sudan	2013	County (80)	State (10)	2010–2012	5 694 045	3
Côte d'Ivoire	2013	District (25)	Region (7)	2005–2013	4 314 015	2
Guinea	2013	Prefecture (38)	Region (8)	2008–2013	3 571 869	2
Togo	2013	District (35)	Region (6)	2005–2013	2 677 790	1
Mauritania	2013	Moughataa (55)	Region (13)	2007–2013	1 468 767	3
Uganda	2014	District (112)	Region (4)	2004–2014	7 581 340	1
Kenya	2014	District (158)	District (158)	2010–2014	1 578 922	1

Data provided in parentheses indicate the number of units included in the analysis.

Abbreviations: DPT, District Prioritization Tool; DRC, Democratic Republic of Congo; LGA, local government area; NA, not applicable; PsA-TT, group A meningococcal polysaccharide–tetanus toxoid conjugate vaccine.

^a^ Pilot implementation.

^b^ Available data were not sufficient to provide final results.

In the majority of countries, standardized meningitis surveillance is conducted nationally. However, for example, in the 11 most southern states of Nigeria, data on suspected meningitis cases were not collected routinely because the risk of epidemic meningitis was deemed very low by national authorities. These areas were therefore not included in the risk analysis. The longest possible time series of standardized epidemiological data were used in individual countries. The meningitis “epidemic season” lasts approximately from December to May, and most cases are reported between January and April [3, 29–31]. Per DPT protocol, surveillance data from full calendar years should be used. However, in Cameroon and Chad, DPT evaluations were conducted in 2011 when meningitis incidence was at its lowest, several months after an intense 2010–2011 epidemic season and a few months before the next one. To reflect the meningitis epidemiological situation as accurately as possible, all data available, including for ongoing year 2011, were included in the analysis, assuming that only a few cases would occur during the remaining months of the year. This assumption was also used in other countries. Nonetheless, epidemiological data rarely spanned beyond 7 years. The time span was particularly short in South Sudan (Table 3), where more informal sources such as funding partner records and unpublished data were used in the model.

District boundary changes also posed a problem. As new districts were created by splitting existing constituencies within the same region, it was often possible to track back their districts of origin and to attribute the data accordingly, using the largest common denominator for the number of districts. These variations were associated with differences in the total duration of case reporting, but the impact on region-level campaigns' phasing was deemed minimal.

Records prior to the implementation of standardized routine surveillance procedures existed in all countries, but they were often event-specific (outbreak data) rather than routine-based and used a spatial reporting scale that was not relevant for the DPT quantitative analysis (eg, available at the state level in Sudan or Nigeria or the regional level in Chad, Cameroon, or Togo). This information was not used for RS computation but informed the qualitative phase of the evaluation.

Consistently low confirmation rates of suspected meningitis cases (data not presented) and missing or limited laboratory data (eg, available at the regional level, or in the catchment area of national or regional laboratories) impeded the computation of the ERI [31]. We relied on historical records to validate the assumption that at least 1 case of meningitis per district and per year was due to NmA when it was the predominant serogroup in the country. The hypothesis did not hold in countries where it was known that other serogroups were also prevalent and where laboratory data were too sparse to ascertain whether NmA was the predominant serogroup, as was the case in the Democratic Republic of Congo, with NmC. The design of a valid introduction strategy for PsA-TT based on existing, retrospective information was therefore not possible under those conditions. We also used information on reactive campaigns with A/C polysaccharide vaccines as a proxy for NmA outbreaks in a district or a region with no known recent history of group C epidemics or identification. This information was obtained from MOHs and partners involved in the implementation of these campaigns, such as the International Coordinating Group on Vaccine Provision, or nongovernmental organizations (eg, Sudan, 2007, in the locality of East Sinnar).

### Outputs: Risk Scores and Categories, Risk Mapping, Performance Flags, and District Profiles

In each country, 1 RS per indicator and per district was computed over the study period for all districts with routine meningitis surveillance. There was a great deal of variation from country to country, and only a few examples are presented for illustrative purposes. The outputs provided are relevant for intracountry prioritization only and may not be used for intercountry comparison.

Except in Ezo district (Western Equatoria region, South Sudan) and Khartoum (Sudan) where CBR scores reached 100, CBR maxima were 75, as in Nigeria, in the local government areas of Bauchi and Katagum (Bauchi state), Yamaltu Deba (Gombe state), and Katsina (Katsina state). In Khartoum, CBR scores of 100 were recorded in the densely populated localities of Jebal Aulya, Karari, and Um Badah with a possibly stronger surveillance system. These localities also had CIR scores ≥50, indicating incidences higher than expected for their population given the national average. In most situations, CIR maxima were 75, capturing facilitating factors of meningitis transmission in a nonspecific manner. Such a value was recorded in the Goundi district of the Mandoul delegation in southern Chad, for instance, which also had an ERI score of 66 (ie, an ERI value of 0.3–0.4; Table 2), indicating that the district experienced a meningitis outbreak every 2–3 years over the study period (Table 3). In all countries, none of the ERI score maxima reached 100—that is, with an epidemic every year or every other year. This finding was surprising in countries such as Ethiopia, known to frequently experience recurring outbreaks in specific areas [10, 31]. In Ethiopia, this could be related to a discrepancy between the geographic scale at which these outbreaks occur and are documented (“woreda,” equivalent of a subdistrict, with populations ranging from 3000 to 600 000) vs the one used for surveillance with standardized, consistent data—hence used as input for the DPT evaluation (“zone,” with populations often >500 000, up to 3 million).

**Figure 3.**
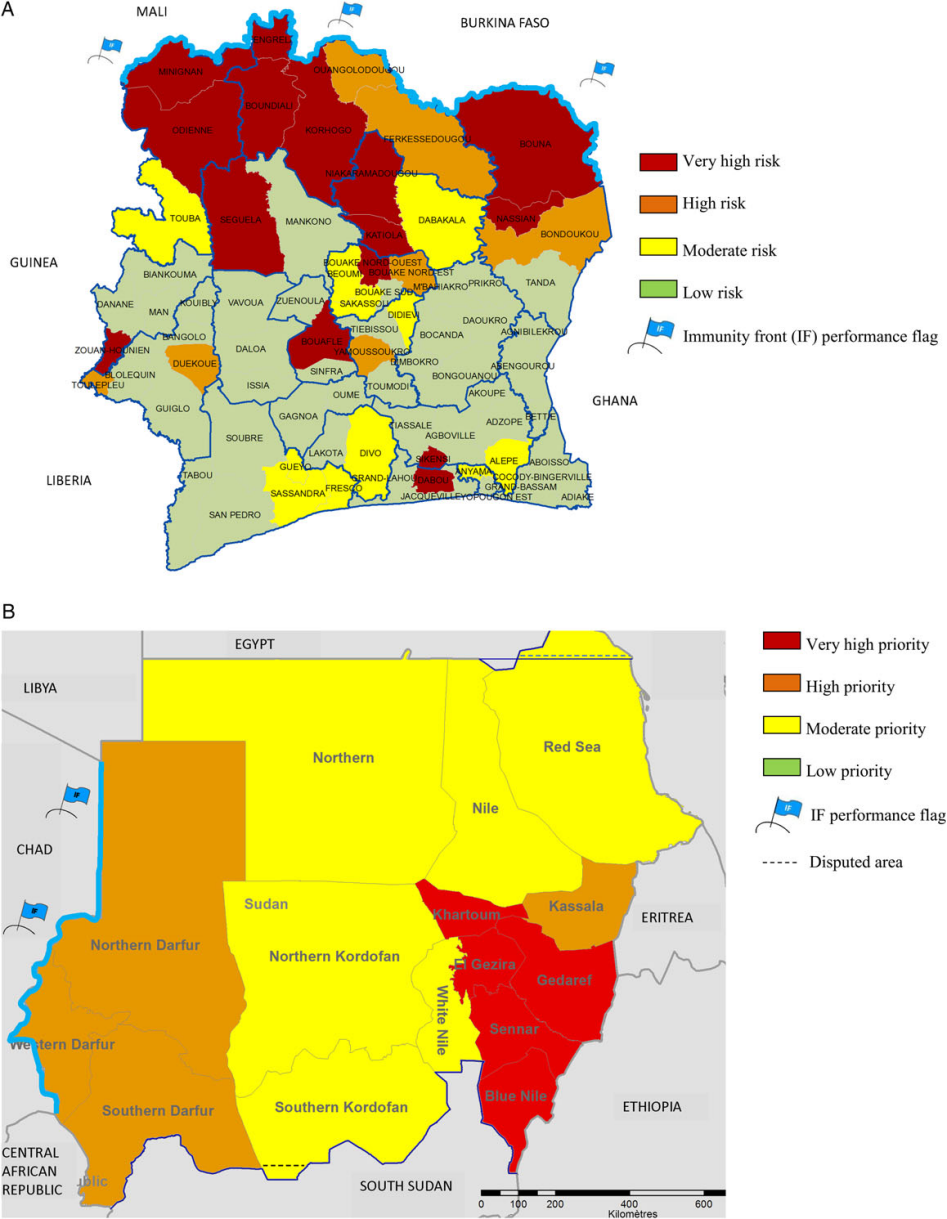
Example of District Prioritization Tool quantitative outputs submitted to local expert review. *A*, District-level risk category in Côte d'Ivoire using 2005–2013 surveillance data. *B*, State-level priority phase in Sudan using 2005–2011 surveillance data. (Ability to conduct the campaigns performance flags were deliberately omitted for the sake of clarity.)

Mapped examples of total RSs by district and associated risk category are provided in Figure 3. Risk mapping was helpful in understanding intracountry risk distribution and determining priority levels to facilitate strategic planning. Maps also highlighted areas where further studies might be useful. For example, some areas yielded untypical results for areas at the fringes of the meningitis belt [1, 22]: some districts of the coastal areas of Cameroon, Côte d'Ivoire, and Guinea had high RSs (see Figure 3 for Côte d'Ivoire) whereas laboratory confirmation rates were low, and in Cameroon and Côte d'Ivoire the temporal distribution of the suspected meningitis cases suggested a different etiology.

Nonetheless, the risk maps were very useful in assessing geographic continuity of the PsA-TT immunization campaigns. The IF being built across the meningitis belt against NmA was an important component of the intracountry priority-setting exercise (Figure 3), as well a useful picture of the situation at the regional level [3]. The majority of data used to assign ACC flags came from reported rather than surveyed vaccine coverage (data not presented), and their high values resulted in most districts being attributed an ACC flag. Such information was seldom used at the stage of priority setting and campaign phasing, but can be useful at a later microplanning phase [10]. District profiles provided a baseline for follow-up and for rapidly monitoring several aspects of the epidemiology of meningitis after the introduction of PsA-TT [10].

### Expert Opinion

Assumptions were discussed and agreed upon with national meningitis experts. They interpreted the results obtained with the DPT and enabled reconciling information from different sources and/or format, for instance, in countries such as Ethiopia or South Sudan where “epidemics” datasets existed but could not be used as input data for the DPT quantitative assessment. The knowledge and reflection of local meningitis experts were critical in informing the risk evaluation. They identified areas with frequent cross-border migrations or population influx that were not reflected in official records (eg, in the mining regions of Guinea) yet represented potential epidemic risk factors and needed to be accounted for in the number of vaccine doses necessary. Local expertise helped to give nuance to the qualitative outputs (eg, “high risk” identified in some coastal areas of Côte d'Ivoire). Local expertise was indispensable to address complex situations aggravating outbreaks and impeding epidemic detection and response, such as conflicts and displaced populations. It also allowed the taking into account of a strong local capacity to detect and contain outbreaks, such as in the state of Al Gezira, Sudan, which on account of this particular strength was scheduled for the second phase of introduction despite an identified high risk. In countries such as Togo, Uganda, or Kenya, local experts also confirmed in which areas mass preventive immunization against NmA would not be relevant. Priorities were adjusted based on these factors, and the list and priority levels of the areas proposed for immunization tailored accordingly. Importantly, engaging national stakeholders throughout the entire risk evaluation process was instrumental in their appropriation and acceptance of the results. This, in turn, appeared to enhance the sustainability and success of DPT-derived vaccine introduction strategies and provided a good opportunity for in-country advocacy for the introduction of the NmA conjugate vaccine [32]. In all countries, DPT evaluations also triggered discussions with national stakeholders on the importance of preparing early for the campaigns, of strengthening meningitis surveillance and data management to better monitor and evaluate the impact of PsA-TT, and of careful outbreak response and case management across the country, including in vaccinated areas where non-A meningococcal serogroups and other pathogens may become the primary cause of meningitis cases.

## CONCLUSIONS

DPT evaluations were done in most of the countries that introduced PsA-TT since 2011, and intracountry priorities were set accordingly. They used an original integrated approach that maximized existing evidence and local expertise to inform vaccine demand and distribution and identify areas where PsA-TT immunization may not be relevant. Evidence-based and reproducible, the DPT accommodated field conditions and countries' specificities. It provided a support for discussions among national stakeholders, but also with international policy makers and donors by providing a regional picture of the expansion of the IF against NmA. DPT risk analyses also supported in-country advocacy for surveillance and vaccination. Despite significant strengths, however, DPT has 2 main limitations: the reliance on epidemiological and microbiological surveillance data and associated data quality issues, and the inclusion of data since the implementation of standardized surveillance for meningitis, spanning a relatively short period of time. We anticipated the limited availability and quality of the data when designing the tool and purposely chose an approach that would make the best of secondary data, in a standardized yet flexible manner that greatly involved local meningitis experts. Although the RIs were conceived to address the characteristics of meningococcal meningitis only, the principles of DPT could be replicated to set priorities for other diseases.
